# Completion of Hepatitis B Vaccination Schedule Among Healthcare Workers in Four Counties in Kenya: A Mixed-Methods Study

**DOI:** 10.3390/vaccines14100851

**Published:** 2026-09-27

**Authors:** Harriet Mirieri, Jeanette Dawa, Dalmas Omia, Rose Jalang’o, Rosalia Kalani, Cynthia Ombok, Sheila Libendi, Wendy Tirop, Elizabeth Kiptoo, Stephen Wandei, Imani Baraka, Salma Swaleh, Joshua Ondiege, Kennedy Oruenjo, Rania A. Tohme, Linus Ndegwa, M. Kariuki Njenga, Gideon O. Emukule, Eric Osoro

**Affiliations:** 1 Washington State University (WSU) Global Health Kenya, Nairobi P.O. Box 72938-00200, Kenya; 2 Institute of Anthropology, Gender and African Studies, University of Nairobi, Nairobi P.O. Box 30197-00100, Kenya; 3 National Vaccines and Immunization Program, Ministry of Health, Nairobi P.O. Box 43319-00100, Kenya; 4 Division of Disease Surveillance and Response, Ministry of Health, Nairobi P.O. Box 30016-00100, Kenya; 5 Department of Health, Nakuru County, Nakuru P.O. Box 2870-20100, Kenya; 6 Department of Health, Kakamega County, Kakamega, P.O. Box 36-50100, Kenya; 7 Department of Health, Mombasa County, Mombasa P.O. Box 81599-80100, Kenya; 8 Department of Health, Siaya County, Siaya P.O. Box 597-40600, Kenya; 9 Division of Viral Hepatitis, U.S. Centers for Disease Control and Prevention, Atlanta, GA 30329, USA; 10 Influenza Division, US Centers for Disease Control and Prevention, Nairobi P.O. Box 606-00621, Kenya; 11 Paul G. Allen School of Global Health, Washington State University (WSU), Pullman, WA 99164-7010, USA

**Keywords:** hepatitis B virus, hepatitis B vaccine, healthcare worker vaccination, multi-dose vaccination, vaccine-preventable diseases, vaccination schedule, coverage, adherence

## Abstract

**Background/Objectives:** Healthcare workers (HCWs) are at an increased risk of hepatitis B virus infection through occupational exposure, yet completion of the recommended hepatitis B vaccination schedule remains suboptimal in Kenya. We assessed vaccination completion, associated factors, motivators, and barriers among HCWs. **Methods:** We conducted a mixed-methods study combining a cross-sectional survey of 1614 HCWs in 178 health facilities across Kakamega, Nakuru, Mombasa, and Siaya counties with nine focus group discussions. The survey was conducted in August–September 2023 and the focus groups during June 2024–January 2025. Mixed-effects logistic regression with a random intercept for health facility was used to identify factors associated with completion of three vaccine doses, and qualitative data were analyzed thematically. **Results:** Among 1583 HCWs with a known vaccination status, 700 (44.2%; 95% CI: 41.8–46.7) reported completing three doses, 410 (25.9%) had received one or two doses, and 473 (29.9%) had never been vaccinated. The main motivator was self-protection (71.7%). Common barriers were vaccine unavailability (57.5%), lack of knowledge (28.8%), the vaccine not being offered on site (28.2%), and cost (17.4%). Completion was more likely among male HCWs, those working in urban areas facilities, and those with 6–10 or 11–20 years of healthcare work experience, and less likely among support staff, administrative staff, clinicians and allied health professionals than among nurses/midwives. **Conclusions:** Fewer than half of HCWs reported completing the three-dose hepatitis B vaccination schedule. Improving vaccine availability, reducing out-of-pocket costs, strengthening dose-reminder systems, and addressing knowledge gaps may improve completion among HCWs in the study counties.

## 1. Introduction

Hepatitis B virus (HBV) infection affects approximately 240 million people globally and is responsible for 1.1 million deaths annually due to complications such as cirrhosis and liver cancer [1]. The burden of HBV infection is particularly high in sub-Saharan Africa, with an estimated prevalence of 5.1% (95% confidence interval (CI): 4.1–6.3) [1]. Healthcare workers (HCWs) are at an increased risk of occupational exposure to HBV from infected blood or contaminated body fluids following needle-stick and sharps injuries [2]. Globally, sharps injuries account for 66,000 cases of HBV infection among HCWs each year [2]. The risk of contracting HBV infection among unvaccinated HCWs after a single exposure ranges from 6 to 30% depending on the infectivity status of the source and the degree of contact with infected blood [3]. A systematic review of studies that were conducted in Africa reported a 6.8% prevalence of HBV infection among HCWs [4], with some studies reporting up to a four times increased risk of HBV infection among HCWs compared to the general population [5,6]. Studies that have been conducted in Kenya found the prevalence of HBV infection among the general population to range between 3 and 8% [7,8]. A separate study among HCWs estimated hepatitis B prevalence to be 4% [9]. These findings demonstrate the continued occupational risk of HBV infection among HCWs. Hepatitis B vaccination is an established preventive intervention and is recommended for health workers at risk of occupational exposure to blood or body fluids [10].

The World Health Organization recommends routine hepatitis B vaccination for HCWs prior to starting clinical duties using a three-dose schedule administered at 0, 1 and 6 months [11]. Where feasible, pre-vaccination serological screening to confirm existing infection or prior immunity is recommended [11]. After completion of the vaccination schedule, serological testing is recommended for high-risk HCWs (those with reasonably anticipated exposure to blood or body fluids in the course of their duties) to confirm protective immunity and additional doses or repeat vaccination is recommended for those that fail to mount an adequate immune response [12]. In Kenya, the Ministry of Health’s National Policy Guidelines for Immunization 2023 recommends hepatitis B vaccination for HCWs at risk of exposure to blood and body fluids using a three-dose schedule administered at 0, 1 and 6 months [13]. In Kenya, however, hepatitis B vaccination is recommended for HCWs at risk of occupational exposure but is not currently a mandatory requirement for HCW employment or clinical practice, contributing to inconsistent uptake across cadres and health facilities [14,15]. Consequently, the majority of HCWs are either not vaccinated against hepatitis B, partially vaccinated, or are unsure about their vaccination status [16,17].

Previous studies in sub-Saharan Africa have reported the high cost of the hepatitis B vaccine, vaccine unavailability and inadequate information regarding the vaccine as the main barriers to uptake. In Kenya, there is limited evidence on adherence to the recommended hepatitis B vaccination schedule and factors influencing uptake among HCWs. The existing studies have mainly been small surveys that were limited to single health facilities or individual counties [14]. Our study sought to address these gaps by examining hepatitis B vaccine uptake and factors associated with completion among HCWs across four diverse counties in rural and urban settings. By employing a mixed-methods approach, our study used qualitative experiences to provide context and a comprehensive understanding of the quantitative findings. Findings from this study may inform context-specific strategies to improve hepatitis B vaccine coverage among HCWs in Kenya. These findings may also support broader efforts aligned with the 2030 hepatitis elimination agenda and Sustainable Development Goal 3.3 [18,19].

## 2. Materials and Methods

### 2.1. Study Population

We conducted this study among HCWs working in 12 sub-counties in four counties in Kenya: Kakamega, Mombasa, Nakuru and Siaya. Based on the 2019 population and housing census data [20], we classified the sub-counties as either urban or rural and purposively selected one-third of sub-counties within each county to obtain seven rural and five urban sub-counties. These sub-counties represent approximately 5% of the national population [20].

The survey was conducted among both clinical and non-clinical health workers in health facilities who had direct contact with patients or potentially infectious surfaces, equipment or materials. Health facility staff who did not regularly have direct contact with patients or potentially infectious surfaces, equipment or materials in the course of their normal duties were excluded from this study.

### 2.2. Study Sites and Design

We implemented a mixed-methods study design combining a cross-sectional baseline survey and focus group discussions (FGDs) targeting HCWs. It was nested within an HCW influenza vaccination project, implemented in collaboration with the Kenya Ministry of Health, to provide a free influenza vaccine to HCWs in the four selected counties and determine rates of acceptance of the influenza vaccine. The baseline survey, which included questions on vaccine-preventable diseases in the workplace, including hepatitis B vaccination status, was conducted during August–September 2023, prior to the influenza vaccination campaign, and leveraged the infrastructure and systematic engagement established by the parent influenza vaccination project.

After the influenza vaccination exercise, we conducted three focus group discussions (FGDs) with HCWs who had received the influenza vaccine (group 1), three FGDs with HCWs who had not received the influenza vaccine (group 2), and three FGDs that included both influenza-vaccinated and unvaccinated HCWs (group 3) to explore their views on HCW vaccination. Although the FGD groupings were based on influenza vaccination status, the discussions also explored attitudes and perceptions towards other vaccines recommended for HCWs, including hepatitis B vaccination.

### 2.3. Sample Size Calculation

This hepatitis B vaccination analysis used the HCW sample recruited for the parent influenza vaccination project; therefore, the sample size was determined by the parent study rather than by a hepatitis B-specific power calculation. The parent study used the standard sample size formula for comparing proportions [21]. Based on prior work among Kenyan HCWs, the parent study assumed that approximately 60–80% of HCWs would report willingness to receive the seasonal influenza vaccine [22]. For program performance, the parent study assumed that influenza vaccination coverage would be 50–70% in the first year and 40–60% in the second year. To detect these differences with 80% power and a two-sided significance level of α = 0.05, the parent study required 354–385 HCWs in each of two comparison groups, corresponding to a minimum of 740–800 participants. To allow comparisons between HCWs working in rural and urban sites, the study targeted at least 800 participants from rural sub-counties and 800 participants from urban sub-counties. The same enrolled sample was used to assess hepatitis B vaccination status and factors associated with completion of the three-dose hepatitis B vaccination schedule.

### 2.4. Sampling

We contacted the departments of health in each county to obtain the list of health facilities in each of the study sub-counties and the number of HCWs within the health facilities. We then stratified the health facilities by ownership (government-owned, private, or faith-based) and by levels (level 2—dispensaries, level 3—health centers, level 4—sub-county hospitals or level 5—county referral hospitals). Using the Kenya Ministry of Health human resource for health norms and standards [23] together with the available county staffing data, we obtained the total facility staffing and used these data to guide expected recruitment per facility level and ownership (Table S1).

Based on the national human resource for health norms and observed staffing patterns, we defined recruitment targets per facility level based on the anticipated number of eligible and willing HCWs available for recruitment per facility level. These were approximately 2 individuals for level 2 health facilities, 7 for level 3 health facilities, 50 for level 4 health facilities and 200 individuals for level 5 health facilities. These estimates represent the recruitment targets per facility level rather than the total facility staffing.

We enrolled an equal number of participants from rural and urban sub-counties, and distributed the study participants across public, private and faith-based organizations proportionate to the number of HCWs in each type of health facility. All health facilities in the study sub-counties were included in the sampling frame. The research assistants visited the health facilities on their list sequentially to enroll as many health workers as possible across different cadres. Once no additional individuals were available or willing to be enrolled in the health facility, the research assistants moved to the next facility on the list until the required sample size for the type of health facility was achieved.

### 2.5. Data Collection

We collected data from consenting participants using a structured survey tool, which was programmed on Research Electronic Data Capture (REDCap) software version 5.24 [24]. The survey tool was pilot-tested for completeness, clarity, logical flow and skip patterns at the Siaya county hospital, in a subset of HCWs who were not included in the study and subsequently modified for the final data collection.

The quantitative tool was administered in-person by trained research assistants who collected data on the HCWs’ sociodemographic characteristics, knowledge of healthcare-associated infections, vaccination status and their willingness to receive vaccines recommended for HCWs including hepatitis B vaccine.

Qualitative data were collected through nine FGDs conducted during June 2024–January 2025. Participants were purposively selected based on their influenza vaccination status to maximize the diversity of vaccination attitudes. Although the primary aim of the FGDs was to explore influenza vaccine uptake, the interview guides included specific questions on hepatitis B vaccination, allowing for exploration of motivators, barriers, and perceptions related to hepatitis B vaccination. The FGDs were conducted among healthcare professionals who participated in the baseline survey of August to September 2023. For the FGDs, we limited participant selection to HCWs who had attained a health-related post-secondary school training/qualification. These HCWs were invited for the FGD at a central place within their counties. The discussions were conducted in Kiswahili or English and audio-recorded with the consent of the participants. A social anthropologist who had not participated in the survey or the influenza vaccination project led the discussions, and field notes were recorded by both the interviewer and the note taker. The FGD interview guide included questions on HCWs’ knowledge and understanding of infectious risks at the workplace, their access to recommended vaccinations and the motivators and barriers influencing uptake of these vaccines (Table S2). FGDs comprised 6–12 participants, and the discussions lasted between 60 and 90 min.

### 2.6. Study Definitions and Terms

Eligible survey participants were adults aged ≥18 years working at a health facility who had direct contact with patients or potentially infectious surfaces, equipment, or materials. Healthcare professionals eligible for FGDs were adult HCWs who had attained a health-related post-secondary school training or qualification. Nurses/midwives were defined as registered or enrolled nurses and midwives providing patient care or related clinical services. Clinicians were defined as clinical officers, doctors, pharmacists, pharmaceutical technologists, dentists or dental technologists. Laboratory staff were defined as laboratory technologists and technicians. An allied health professional was defined as either a health records officer, public health officer, nutritionist, social worker, mortician, physiotherapist, radiographer, occupational therapist, plaster technician, theatre technician or orthopedic technologist [25]. Administrative staff were defined as personnel performing managerial or clerical functions, and they were included in this study only if their duties involved direct patient interaction or exposure to clinical environments or potentially infectious materials. These included receptionists working at registration desks, clerks handling patient files in wards or clinical areas, administrators, and revenue officers. Support staff were non-clinical hospital personnel whose duties involved direct or indirect exposure to patients, patient-care areas, or potentially infectious surfaces, equipment or materials and were exposed through handling contaminated materials, cleaning patient-care areas, transporting patients, or working in environments where exposure to infectious materials may occur. These included cleaners, patient attendants, laundry attendants, porters, and mortuary attendants. Body mass index (BMI) was defined as underweight (≤18.4 kg/m^2^), normal weight (18.5–24.9 kg/m^2^), overweight (25.0–29.9 kg/m^2^), or obese (≥30 kg/m^2^) [26]. Primary health facilities comprised level 2 (dispensaries) and level 3 health facilities (health centers) while referral health facilities included level 4 (sub county referral hospitals) and level 5 health facilities (county referral hospitals). Minimal patient contact referred to HCWs who did not have contact with patients and whose routine work was not in a clinical area; moderate patient contact referred to HCWs who had contact that was not physical but would allow the acquisition or transmission of diseases that are spread at short range by respiratory means, while significant patient contact referred to direct physical contact with patients or with infectious materials, including surfaces or equipment contaminated by infectious materials [11].

### 2.7. Data Analysis

Quantitative data were cleaned and analyzed using R software version 4.4.3. Descriptive statistics (medians and interquartile ranges [IQR]) were used to summarize the participant’s age and average years worked in healthcare. Other characteristics of the study population were presented using proportions and 95% confidence intervals (CIs). Categorical variables were compared using the chi-square test.

Participants were categorized into three groups based on self-reported hepatitis B vaccination status: (1) those that had completed the recommended three-dose schedule, (2) received one or two doses of the vaccine (partial-uptake) or (3) never received hepatitis B vaccine. Cadres were classified either as nurse/midwife, clinician, laboratory staff, allied health professional, support staff or administrative staff to minimize loss of contextual differences between cadres that may occur with broad clinical versus non-clinical classifications.

Our primary analysis compared the complete hepatitis B vaccination schedule with incomplete vaccination (partial uptake or no uptake combined), as completion of the recommended three-dose schedule was the primary outcome of interest. To assess whether factors associated with uptake (initiation) differ from those associated with adherence (completion), we conducted sensitivity analyses comparing (1) complete versus partial uptake, and (2) complete versus no uptake. Comparisons between these groups were summarized using proportions and 95% CIs and assessed using regression models as described below.

We used generalized linear mixed-effects models with a binomial family and logit link to estimate odds ratios. We accounted for clustering of HCWs within health facilities by including a random intercept for the health facility. Multivariable mixed-effects logistic regression models were used were used to identify factors independently associated with complete vaccine uptake, while adjusting for potential confounders. Variables with a *p*  ≤  0.25 in the univariable analysis and those considered a priori to be important confounders based on existing knowledge were included in the initial multivariable model [27]. The cadre and previous experience of adverse reactions were retained a priori in all models since the occupational role and previous adverse reactions may influence hepatitis B vaccine uptake. Backward elimination guided by the Akaike information criterion (AIC) was used to obtain a parsimonious final model. Statistical significance was set at a *p*-value < 0.05. Multicollinearity was assessed using the variance inflation factor (VIF). Variables with evidence of collinearity were not included simultaneously in the multivariable model. We tested pre-specified two-way interaction terms between variables in the final model; interactions with *p* ≥ 0.05 were not retained.

For the qualitative data, a research assistant transcribed the audio recordings, and the qualitative researcher cross-checked the transcripts against the audio records to ensure the accuracy and quality of transcribed data. Qualitative data were analyzed using inductive thematic analysis, allowing the themes to emerge from the data. Transcripts were coded and organized using NVivo version 12. Emerging themes related to barriers and motivators were identified. The qualitative findings were used to provide context for the quantitative results.

### 2.8. Ethical Considerations

This study protocol was approved by the African Medical and Research Foundation (AMREF) Ethical and Scientific Review Committee (ESRC) (AMREF-ESRC P1426/2023), approved on 20 June 2023; Coast General Teaching and Referral Hospital Ethical Review Committee (ERC-CGH/MSc/VOL.1); and Kakamega County General Hospital Ethics Review Committee (ERC/209-07/2023). Reliance approval was provided by the Washington State University Institutional Review Board based on in-country ethical approvals. This activity was reviewed by the CDC and was conducted consistently with applicable federal law and CDC policy as provided for in the Code of Federal Regulations (45 C.F.R part 46 and 21 C.F.R. part 56). Administrative approval was provided by the National Commission for Science, Technology and Innovation and the departments of health of all participating counties. Written consent was obtained from survey and focus group discussion participants before initiation of any study activity.

## 3. Results

### 3.1. Characteristics of Participants Enrolled in This Study

We enrolled 1614 HCWs from 178 health facilities from August to September 2023. Of these, 1583 (98.1%) were included in the analytic sample. The 31 (1.9%) HCWs who were unsure of their hepatitis B vaccination status were excluded from the analysis. Percentages presented in the results are based on the analytic sample size (N = 1583), unless otherwise specified. Participant inclusion in the analytic sample is summarized in Supplementary Figure S1.

A total of 996 (62.9%) participants were female, 517 (32.7%) were nurses/midwives, and 1013 (64.0%) had attained a diploma-level education (Table 1). The majority of the participants reported significant patient contact (1291; 81.6%). The median age of the participants was 33 years (IQR: 28–40), and the median duration of work experience was 6 years (IQR: 3–10). Only 199 (12.6%) participants had an underlying medical condition, of which the most common were asthma (39/199, 19.6%), hypertension (32/199, 16.1%), diabetes (25/199, 12.6%) and chronic cardiac disease (13/199, 6.5%). A total of 1077 (68.0%) participants worked in public health facilities, 1023 (64.6%) were from referral health facilities and 793 (50.1%) worked in urban health facilities.

Among the 1583 participants, 700 (44.2%) reported completing the recommended three-dose hepatitis B vaccination schedule, 410 (25.9%) reported receiving one or two doses, and 473 (29.9%) reported never receiving hepatitis B vaccine.

### 3.2. Reasons for Receiving Hepatitis B Vaccine Among Participants Who Reported Completing the Recommended Hepatitis B Vaccination Schedule (N = 700)

Self-protection, at 71.7% (95% CI: 68.3–74.9), was the most commonly reported reason for receiving the hepatitis B vaccine (Table 2).

“I think vaccination is very necessary, considering the fact that we get in contact with vulnerable clients. So, it’s good we are vaccinated so that we cannot really transmit to vulnerable clients or even just normal clients.”

(FGD Group 1)

Overall, 32.1% (95% CI: 28.8–35.7%) of respondents had received the hepatitis B vaccine because it was a requirement or was recommended during training. Some HCWs received the vaccine because it was a mandatory requirement or was recommended at the workplace: 17.7% (95% CI: 15.1–20.7%).

“Some facilities go an extra mile to support their staff, just to ensure they are vaccinated. For those accredited facilities, the auditors check to ensure that everybody gets vaccinated. So, some facilities go an extra mile to do it for their staff. Also, there are some institutions, learning institutions, they try to push that. Even in colleges, they try to push for hepatitis B vaccination.”

(FGD Group 1)

Protecting family members (13.9%, 95% CI: 11.6–16.6%) and protecting patients (8.9%, 95% CI: 7.0–11.2%) were also cited as enablers for vaccination.

“I think healthcare workers also require vaccination because apart from us being at the greatest risk, we also need to protect our own because we also have families, we have kids, we have partners. So when we are vaccinated, we also protect others, including our families and friends.”

(FGD Group 2)

Other reported reasons included the vaccine being offered for free of charge (9.7%, 95% CI: 7.8–12.1%), ease of access (9.0%, 95% CI: 7.1–11.3%), requirements or recommendations from the government or Ministry of Health (8.6%, 95% CI: 6.7–10.9%) and prevention of complications or severe disease (7.9%, 95% CI: 6.1–10.2%).

### 3.3. Reasons for Incomplete or No Uptake of Hepatitis B Vaccine Among Participants Who Reported Partial or No Hepatitis B Vaccination

Among the 883 participants who reported either partial hepatitis B vaccination (n = 410) or no vaccination (n = 473), vaccine unavailability, at 57.5% (95% CI: 54.2–60.8%), was the most commonly reported barrier, with the reason being more frequently cited among those with partial vaccination (64.6%, 95% CI: 59.9–69.1%) compared to those that had never received the vaccine (51.4%, 95% CI: 46.9–55.8%) (Table 3).

“Well, from past experience hepatitis B used to be erratic. Availability was erratic. There are times it is there, other times it’s not there. You send staff to go and have it and they find it’s out of stock. So, availability was a challenge.”

(FGD Group 1)

HCWs expressed concerns that whenever the hepatitis B vaccine was availed, it often arrived at their facilities close to the expiration date. This led to perceptions that HCWs were not prioritized for recommended vaccines and that near-expiry vaccines were provided mainly to avoid wastage, contributing to safety concerns.

“Currently, hepatitis B is not available every time. And I remember there was a time when the vaccine for hepatitis B was about to expire. They decided that instead of the medicine to expire, they inject it to people so that they don’t expire yet it cost a lot of money. It was brought and health workers were injected. There was someone going around the facility during the procedure [administering the vaccine]. Simply because they were about to expire.”

(FGD Group 1)

“Actually, what happens, they bring the vaccines when they are about to expire. They promise to bring a second dose, they don’t come. So, we are not regarded as first priority in the vaccines as healthcare workers. They only bring them when they are about to expire and they don’t want to waste”

(FGD Group 1)

Other commonly cited reasons included not being offered the hepatitis B vaccine (28.2%, 95% CI: 25.3–31.3%) and lack of knowledge about the vaccine (28.8%, 95% CI: 25.9–31.8%).

“I think there is a lot of assumption that medics or anybody working in the hospital facility is aware of everything. So, I think allowing sensitization programs in the facility is also very important. So, that you don’t just come up with a memo, and you say, we have a vaccine for hepatitis B. I think you sensitize people, tell them the risks, tell them how important it is. Because if you don’t do that, they won’t know [there] is a vaccination program going in the facility”

(FGD Group 1)

Some participants reported that the cost of the vaccine was prohibitive as they had to pay out of pocket and the health insurance did not cater for the costs. Cost of receiving the vaccine was cited as a barrier by 17.4% of participants (95% CI: 15.1–20.1%). It was more frequently reported among those with partial vaccination (19.8%, 95% CI: 16.2–23.9%) compared to those who had never been vaccinated (15.4%, 95% CI: 12.5–19.0%).

“The challenge is the cost for hepatitis B, because you have to get the three [doses]. Because you have to pay for yourself. The employer doesn’t cover the costs. And the insurance cover too doesn’t cover it. So, it’s only out of pocket. If you are getting it from a government facility, the charges are from around 680 shillings [$5.3] per dose. And you have to know where to find it. And then others, it goes up to 2000 [shillings] [$15.5]. It depends. So, you have to know where it’s cost effective.”

(FGD Group 3)

In some private health facilities, although the cost of the vaccine was shared between the employer and the HCW, some HCWs still found the cost unaffordable.

“In our facility, we cost share with the employer. They can pay half, and then you pay the half. So, for other people, they just feel it’s too much for them. They never get the vaccine.”

(FGD Group 2)

The hepatitis B vaccine is only available at select health facilities, and most times, the HCW was required to travel to other health facilities to access the vaccine. The associated transport costs were barriers to vaccination.

“Where I work, they are not readily available [hepatitis B vaccine]. They are there occasionally. Sometimes it is available, sometimes it is not. You just go and ask, if it is available, they will give you, if it is not there they will refer you to somewhere else. So, and that actually increases the cost in terms of time but also associated transport…”

(FGD Group 2)

The HCWs emphasized that inclusion of the hepatitis B vaccination in the health insurance schemes would improve uptake.

“The healthcare worker would be comfortable if the employer can convince us that the insurance we have can still cater for it… that we can go and get the vaccine from a private [facility] and use our insurance to cover for it. I think that kind of arrangement the health workers would consistently take it”

(FGD Group 1)

Among participants who had been partially vaccinated against hepatitis B, 10.0% (95% CI: 7.5–13.3%) indicated a lack of a provider or program-led follow-up system for remaining doses (including reminders for subsequent doses and tracking of defaulters), unlike the childhood immunization program, as a reason for non-completion. HCWs reported that unlike the childhood routine vaccination services, HCW vaccination lacked follow-up for subsequent doses, proper documentation and a structured system of reminders. The physical cards, which sometimes were provided as proof of vaccination, were often misplaced, and there was no digital tracking system to ensure continuity.

“How I wish we get those cards with reminders that the next day is when you are due for vaccination. Then we get a way to be reminding the healthcare workers that your next vaccine is due on this day at this time just like mothers have always been reminded about their days so that we don’t miss out and also know the importance of this vaccine”

(FGD Group 1)

Other reasons, each reported by fewer than 5% of participants, included perceptions that vaccination was unnecessary, concerns about administration or vaccine safety, time constraints, and low perceived risk; the complete distribution is shown in Table 3.

### 3.4. HCW Perceptions on Access to Hepatitis B Vaccine

More than half of the respondents, 60.8% (95% CI: 58.3–63.2), perceived access to hepatitis B vaccination services as either difficult or very difficult (Table S3). This was more commonly reported among individuals who had never been vaccinated (74.6%, 95% CI: 70.4–78.6%) compared to those who had completed the full vaccination schedule (47.4%, 95% CI: 43.7–51.2%) (Figure 1). Conversely, participants who had been fully vaccinated described access to the vaccine as either easy or very easy (37.9%; 95% CI: 34.2–41.6%) compared to only 6.9% (95% CI: 4.8–9.7%) of those who had never received the vaccine. There was a statistically significant association between access to vaccination services and vaccination status (*p* < 0.001).

### 3.5. Multivariable Analysis of Factors Associated with Completion of the Multi-Dose Hepatitis B Vaccine Schedule Among HCWs Enrolled in the Study

From the results of the univariable mixed-effects regression models comparing complete hepatitis B vaccine uptake versus partial or no uptake combined as the outcome variable, while accounting for health facility clustering, the sex, age, education, number of years worked in healthcare, level of physical contact with patients, health facility ownership type, rural–urban classification of sub-counties where HCW worked, health facility level and county met the threshold for inclusion in the multivariable model at *p*  ≤  0.25. The cadre and previous experience of adverse reactions following vaccination were retained a priori (Table S4).

The county was significantly associated with completion in the univariable analysis (*p* < 0.001; Table S4) but had a strong association with the rural–urban classification and was therefore not retained in the adjusted model. In the adjusted final model, male HCWs had higher odds of completing the hepatitis B vaccination schedule compared to females (adjusted odds ratio (aOR): 1.7; 95% CI: 1.4–2.2) (Table S5) (Figure 2). HCWs aged 40–49 years had lower odds of completing the vaccination schedule compared to those aged 30–39 years (aOR = 0.6, 95% CI: 0.4–0.9). We observed differences in cadres, where support staff (aOR = 0.2, 95% CI: 0.2–0.4), administrative staff (aOR = 0.3, 95% CI: 0.2–0.4), and allied health professionals (aOR = 0.5, 95% CI: 0.43–0.7) had lower odds of full vaccination compared to nurses. Clinicians had slightly lower odds of completing the vaccination schedule compared to nurses (aOR = 0.7, 95% CI: 0.5–0.9). There was no evidence of a difference between laboratory staff and nurses/midwives (Figure 2).

Compared to HCWs with 0–5 years of experience of working in healthcare, those with 6–10 years of experience (aOR = 1.4, 95% CI: 1.0–2.0) and 11–20 years of experience (aOR = 1.9, 95% CI: 1.2–3.0) had higher odds of completing the hepatitis B vaccination schedule. HCWs working in health facilities based in urban areas had about twice the odds of completing the vaccination schedule compared to those in rural areas (aOR = 2.2, 95% CI: 1.5–3.1). None of the interaction terms between variables in the final models were statistically significant.

### 3.6. Sensitivity Analysis

In the sensitivity analysis comparing complete with partial uptake, the male sex and urban location were associated with higher odds of completion, while allied health professionals and support staff had lower odds of completion than nurses/midwives after accounting for clustering within health facilities (Table S6).

In the analysis comparing complete with no vaccination, the male sex and urban location were associated with higher odds of completion. Administrative staff, clinicians, allied health professionals, and support staff had lower odds of completion than nurses/midwives. HCWs with 6–10 years of healthcare work experience and those classified as obese also had higher odds of completion (Table S7).

## 4. Discussion

We found that fewer than half of healthcare workers in the four selected counties in Kenya reported completing the three-dose hepatitis B vaccination schedule. The main motivators for vaccine uptake were self-protection and mandatory requirements or recommendations by the employer or during training. In contrast, common barriers to vaccine uptake were vaccine unavailability, high cost of the vaccine, lack of reminders and insufficient knowledge or awareness about the vaccine. Workplace-based vaccination and provision of the vaccine at no cost were associated with easier vaccine access. In addition, sex, age, cadre, years of experience and rural–urban location were independently associated with completion of the hepatitis B vaccination schedule.

Our self-reported estimate of complete hepatitis B vaccination was higher than the pooled estimate reported in an African systematic review and higher than estimates reported in selected studies from other African settings [28,29,30]. However, these comparisons should be interpreted cautiously because prior studies varied in sampling frame, facility type, study period, and ascertainment of vaccination status. Our estimate was similar to estimates from two referral-facility studies in Kenya [9,14], but our study included HCWs from rural and urban areas and from primary and referral facilities. Despite this broader sampling frame, complete hepatitis B vaccination remained suboptimal.

Almost 56% of participants reported no or incomplete hepatitis B vaccination. Incomplete or non-uptake of the hepatitis B vaccine was mainly associated with health system barriers including availability and cost of the vaccine. Among the partially vaccinated HCWs, the lack of follow-up mechanisms for the subsequent doses was a hindrance to vaccination. These findings are similar to other studies that were conducted in sub-Saharan Africa, which reported vaccine unavailability and cost of the vaccine as the main barriers to hepatitis B vaccine uptake [28,29,31]. In our qualitative data, participants contrasted HCW hepatitis B vaccination with routine childhood immunization services, describing HCW vaccination as less consistently organized around vaccine availability, documentation, reminders for subsequent doses, and follow-up. Participants also reported that vaccines were sometimes offered close to expiry, which they interpreted as a sign that HCWs were not prioritized and which contributed to concerns about vaccine quality, safety, or effectiveness. Consistent with WHO guidance on health-worker vaccination programs, reliable vaccine supply, reduced out-of-pocket costs, workplace-based vaccination, documentation, and reminder or recall systems may improve hepatitis B vaccine completion among HCWs [11].

Completion of the three-dose hepatitis B vaccination schedule was 63.2% among laboratory staff, 54.4% among nurses/midwives, and 52.3% among clinicians. Although higher than in several non-clinical cadres, completion remained suboptimal among HCWs with occupational exposure [11]. Administrative staff, clinicians, allied health professionals and support staff were less likely to have completed hepatitis B vaccination compared to nurses. These cadre differences may partly reflect differences in vaccine awareness, perceived occupational risk, and opportunities to access vaccination. In our study, insufficient knowledge was a main barrier to vaccination, especially among non-clinical staff, aligning with evidence from sub-Saharan Africa [30,32,33]. The differences in vaccine uptake among cadres could also be attributed to prioritization of clinical HCWs for vaccination and vaccination-related communication and training, leaving limited opportunities for non-clinical staff to be trained on vaccines and get vaccinated [34]. There is a need to address vaccine knowledge gaps and disparate uptake between clinical and non-clinical staff. Training across HCW cadres and inclusive vaccination programs that provide access to clinical and non-clinical staff with occupational exposure to blood or body fluids may improve hepatitis B vaccine uptake [11].

Male HCWs in our study had 1.8 times the odds of completing the recommended hepatitis B vaccine schedule compared to their female counterparts. This finding differs from similar studies that have been conducted in low- and middle-income countries, which reported higher vaccine uptake among female HCWs [35,36,37]. In these studies, higher uptake among females was attributed to better health-seeking behaviors, which contribute to higher vaccine uptake [38]. In our setting, the observed differences by sex may suggest underlying differences in occupational roles, exposure patterns or access to vaccination across workstations. Unlike in some childhood immunization contexts where sex-based immunization prioritization has been reported, this was not a feature of the hepatitis B vaccination schedule among HCWs in our context [39,40]. Further research is needed to better understand the sex differences in hepatitis B vaccine uptake among HCWs.

HCWs who worked in urban sub-counties in our study had higher odds of vaccination compared to those who worked in rural sub-counties. These findings have also been reported in other studies [41,42]. This could be explained by more sensitization and the ease of access to hepatitis B vaccination services in urban areas; consistent with our qualitative findings, HCWs described erratic supply, stock-outs, near-expiry stock, and having to travel to other facilities—at additional transport cost—to obtain the vaccine [42].

HCWs with 6–10 and 11–20 years of experience in healthcare had higher odds of completing hepatitis B vaccination compared to those with less than 6 years. Our finding is consistent with a systematic review of African studies, which reported that HCWs with ≥10 years of experience were more likely to complete vaccination than those with less work experience [29]. This association may be related to the differences in occupational risk exposure over time and awareness on the availability of the hepatitis B vaccine during employment. Greater cumulative opportunities to access vaccination over time may also contribute to the higher completion observed among HCWs with longer work experience.

A key strength was the inclusion of different HCW cadres, including clinical and non-clinical personnel with occupational exposure recruited from multiple facility levels across rural and urban sub-counties. Despite these strengths, a limitation of this study is that the hepatitis B vaccination status was self-reported, as we did not verify vaccination documentation or check antibodies against the hepatitis B surface antigen to confirm protection. This may have introduced misreporting or recall bias, leading to inaccurate vaccination coverage estimates. Another limitation is that the study was limited to sub counties that were purposively selected within the four counties and may not be nationally representative. In addition, recruitment of HCWs at the health facility level was conducted sequentially based on willingness and availability until the recruitment targets were achieved. Selection bias cannot be ruled out since HCWs that were not available or were unwilling to participate may be different from those that were enrolled in the study. A further limitation of the mixed-methods design is that the FGDs were conducted after the survey and were organized by influenza vaccination status rather than hepatitis B vaccination status. The qualitative findings therefore provide context for the quantitative findings but were not designed to explain participant-level patterns of hepatitis B vaccination.

## 5. Conclusions

In this four-county sample of Kenyan HCWs, fewer than half reported completing the recommended three-dose hepatitis B vaccination schedule. Completion varied by sex, cadre, years of healthcare work experience and rural–urban location. These differences may partly reflect disparities in vaccine access and awareness, alongside other factors. Strategies adapted from routine immunization systems, including reliable vaccine supply, reduced out-of-pocket costs, workplace-based vaccination, documentation, reminders for subsequent doses, and targeted education for both clinical and non-clinical staff, may improve hepatitis B vaccine completion among HCWs. Improving hepatitis B vaccination among healthcare workers would contribute to preventing occupational HBV infections and support broader efforts to eliminate hepatitis B as a public health problem.

## Figures and Tables

**Figure 1:**
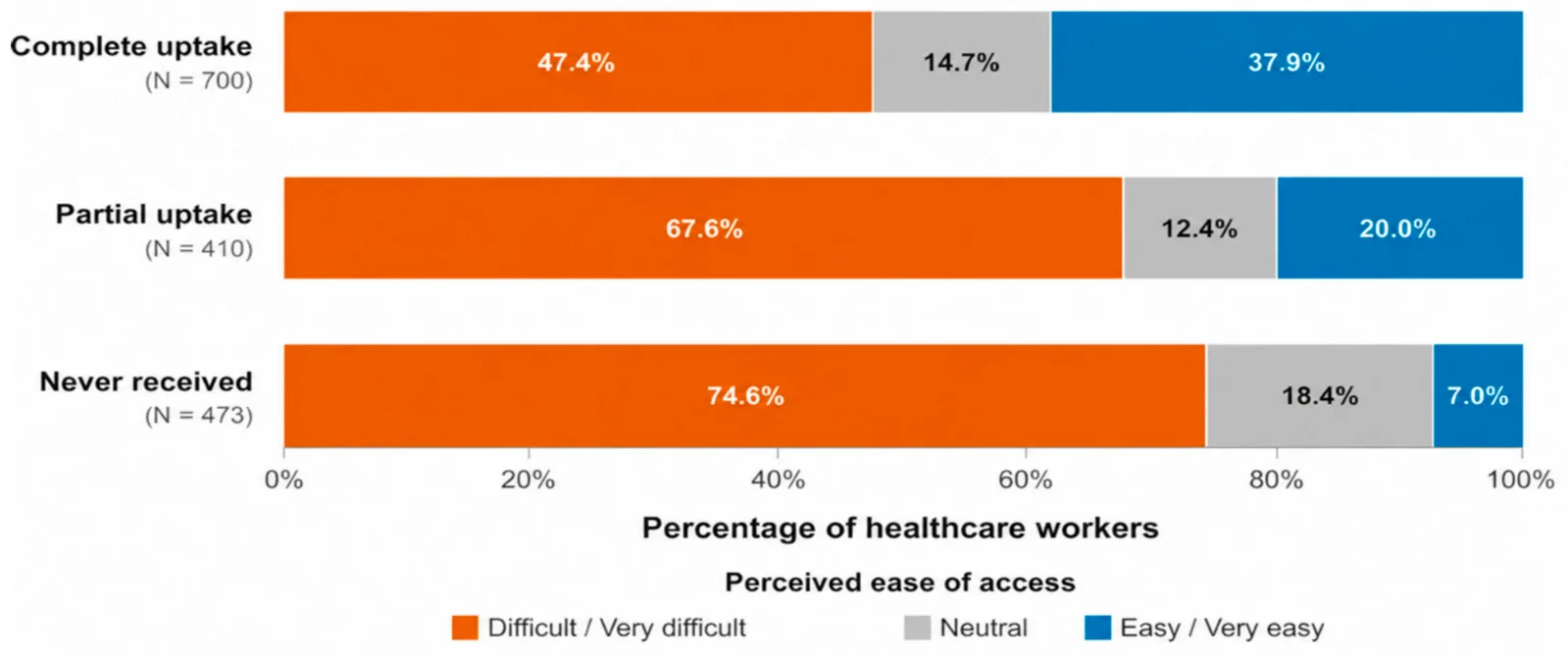
Perceived ease of access to hepatitis B vaccination services among healthcare workers in Kenya, 2023.

**Figure 2:**
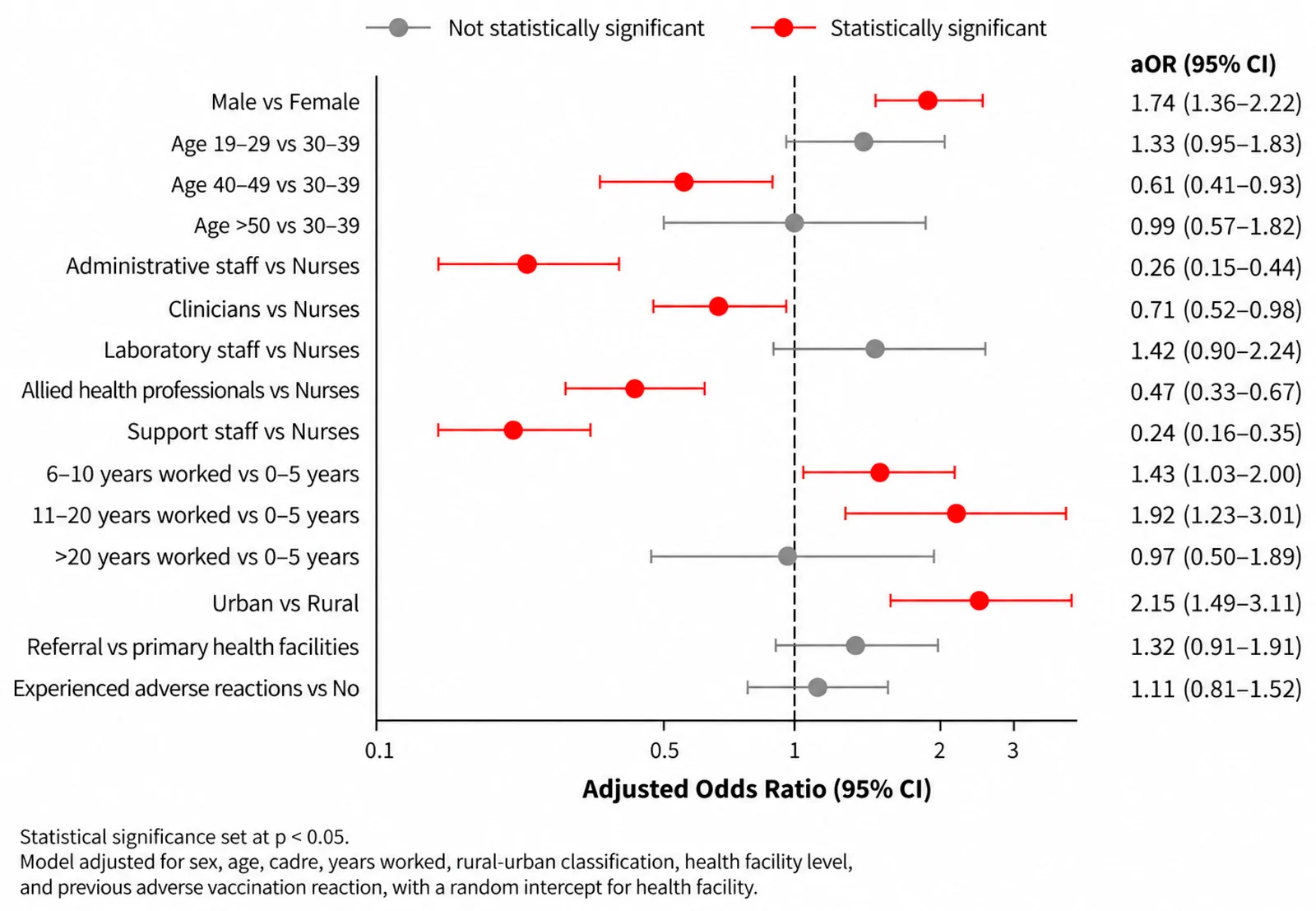
Forest plot of factors associated with completion of the multidose hepatitis B vaccination schedule among healthcare workers in Kenya, 2023 (comparing completed vaccine schedule to partial uptake or no vaccine uptake combined).

**Table 1:** Sociodemographic characteristics of participants enrolled in this study by hepatitis B vaccination status, Kenya 2023.

Characteristic	Overall N = 1583 n (%)	Complete Uptake of Hepatitis B Vaccine N = 700 n (%)	Partial Uptake of Hepatitis B Vaccine N = 410 n (%)	Never Received Hepatitis B Vaccine N = 473 n (%)	*p*-Value ^a^
**Sex**					**<0.001**
Female	996 (62.9)	387 (55.3)	271 (66.1)	338 (71.5)	
Male	587 (37.1)	313 (44.7)	139 (33.9)	135 (28.5)	
**Age**					
Median (IQR)	33 (28–40)	32 (28–39)	33 (28–40.8)	34 (28–42)	**0.039 ^d^**
19–29	543 (34.3)	260 (37.1)	128 (31.2)	155 (32.8)	**0.025**
30–39	614 (38.8)	278 (39.7)	165 (40.2)	171 (36.2)	
40–49	257 (16.2)	90 (12.9)	77 (18.8)	90 (19.0)	
>50	169 (10.7)	72 (10.3)	40 (9.8)	57 (12.1)	
**Cadre**					
Nurse/midwife	517 (32.7)	281 (40.1)	152 (37.1)	84 (17.8)	**<0.001**
Allied health professionals ^b^	245 (15.5)	92 (13.1)	74 (18.0)	79 (16.7)	
Clinician ^c^	310 (19.6)	162 (23.1)	81 (19.8)	67 (14.2)	
Support staff	278 (17.6)	63 (9.0)	56 (13.7)	159 (33.6)	
Administrative staff	108 (6.8)	23 (3.3)	22 (5.4)	63 (13.3)	
Laboratory staff	125 (7.9)	79 (11.3)	25 (6.1)	21 (4.4)	
**Highest level of completed education**					**<0.001**
High school certificate or less	234 (14.8)	49 (7.0)	43 (10.5)	142 (30.0)	
Diploma	1013 (64.0)	472 (67.4)	287 (70.0)	254 (53.7)	
Bachelor’s degree	301 (19.0)	153 (21.9)	75 (18.3)	73 (15.4)	
Post-graduate degree	35 (2.2)	26 (3.7)	5 (1.2)	4 (0.8)	
**Number of years worked in healthcare**					
0–5	774 (48.9)	323 (46.1)	182 (44.4)	269 (56.9)	**0.003**
6–10	423 (26.7)	197 (28.1)	126 (30.7)	100 (21.1)	
11–20	256 (16.2)	120 (17.1)	64 (15.6)	72 (15.2)	
>20	130 (8.2)	60 (8.6)	38 (9.3)	32 (6.8)	
**Level of physical contact with patients**					**<0.001**
Minimal contact	42 (2.6)	17 (2.4)	5 (1.2)	20 (4.2)	
Moderate contact	250 (15.8)	82 (11.7)	56 (13.7)	112 (23.7)	
Significant contact	1291 (81.6)	601 (85.9)	349 (85.1)	341 (72.1)	
**Had underlying medical condition**					
Reported yes	199 (12.6)	83 (11.9)	47 (11.5)	69 (14.6)	0.300
Reported no	1384 (87.4)	617 (88.1)	363 (88.5)	404 (85.4)	
**Health facility type ^f^**					**<0.001**
Public	1077 (68.1)	440 (62.9)	302 (73.7)	335 (71.0)	
Private	435 (27.5)	233 (33.3)	88 (21.5)	114 (24.2)	
FBO	69 (4.4)	26 (3.7)	20 (4.9)	23 (4.9)	
**BMI**					
Underweight (≤18.4)	28 (1.8)	12 (1.7)	8 (2.0)	8 (1.7)	**0.024**
Normal (18.5–24.9)	648 (40.9)	265 (37.9)	156 (38.0)	227 (48.0)	
Overweight (25.0–29.9)	569 (35.9)	262 (37.4)	153 (37.3)	154 (32.6)	
Obese ≥ 30	338 (21.4)	161 (23.0)	93 (22.7)	84 (17.8)	
**Experienced adverse reactions following vaccination ^e^**					0.500
Yes	248 (15.7)	117 (16.7)	63 (15.4)	68 (14.4)	
No	1335 (84.3)	583 (83.3)	347 (84.6)	405 (85.6)	
**Rural–urban classification of sub-counties where HCW works**					**<0.001**
Rural	790 (49.9)	261 (37.3)	231 (56.3)	298 (63.0)	
Urban	793 (50.1)	439 (62.7)	179 (43.7)	175 (37.0)	
**Health facility level**					**<0.001**
Primary health facilities	560 (35.4)	198 (28.3)	163 (39.8)	199 (42.1)	
Referral health facilities	1023 (64.6)	502 (71.7)	247 (60.2)	274 (57.9)	
**County**					**<0.001**
Siaya	536 (33.9)	193 (27.6)	143 (34.9)	200 (42.3)	
Kakamega	281 (17.8)	96 (13.7)	84 (20.5)	101 (21.4)	
Mombasa	362 (22.9)	230 (32.9)	82 (20.0)	50 (10.6)	
Nakuru	404 (25.5)	181 (25.9)	101 (24.6)	122 (25.8)	

^a^ *p*-value comparing hepatitis B vaccination status categories. ^b^ Health records officers, public health officers, nutritionists, social workers, morticians, physiotherapists, radiographers, occupational therapists, plaster technicians, theater technicians, orthopedic technologists. ^c^ Clinical officers, doctors, pharmacists/pharmaceutical technologists, dentists/dental technologists. ^d^ Kruskal–Wallis test *p*-value. Statistically significant differences may reflect small distributional differences, which may not be clinically meaningful given the large sample size. ^e^ Self-reported history of an adverse reaction following receipt of any vaccine, not specific to hepatitis B vaccine. Percentages may not sum to 100 due to rounding. ^f^ Health facility type was missing for two participants.

**Table 2:** Reasons for receiving hepatitis B vaccine among healthcare workers who reported completing the recommended hepatitis B vaccination schedule, Kenya 2023.

Why Did You Receive the Hepatitis B Vaccine?	Kakamega N = 96 n (%, 95% CI)	Mombasa N = 230 n (%, 95% CI)	Nakuru N = 181 n (%, 95% CI)	Siaya N = 193 n (%, 95% CI)	Total N = 700 n (%, 95% CI)
To protect myself	75 (78.1, 68.9–85.2)	166 (72.2, 66.1–77.6)	131 (72.4, 65.4–78.4)	130 (67.4, 60.5–73.6)	502 (71.7, 68.3–74.9)
It is/was a requirement or recommended during my training	26 (27.1, 19.2–36.7)	29 (12.6, 8.9–17.5)	74 (40.9, 34.0–48.2)	96 (49.7, 42.8–56.7)	225 (32.1, 28.8–35.7)
It is/was a requirement or recommended at my workplace/by my employer	3 (3.1, 1.1–8.8)	79 (34.3, 28.5–40.7)	17 (9.4, 5.9–14.5)	25 (13.0, 8.9–18.4)	124 (17.7, 15.1–20.7)
To protect my family	7 (7.3, 3.6–14.2)	19 (8.3, 5.4–12.5)	51 (28.2, 22.1–35.1)	20 (10.4, 6.8–15.5)	97 (13.9, 11.6–16.6)
It is/was offered for free	4 (4.2, 1.6–10.2)	16 (7.0, 4.3–11.0)	26 (14.4, 10.0–20.2)	22 (11.4, 7.6–16.7)	68 (9.7, 7.8–12.1)
It is/was readily accessible	5 (5.2, 2.2–11.6)	11 (4.8, 2.7–8.4)	18 (9.9, 6.4–15.2)	29 (15.0, 10.7–20.7)	63 (9.0, 7.1–11.3)
To protect my patients	21 (21.9, 14.8–31.1)	16 (7.0, 4.3–11.0)	13 (7.2, 4.2–11.9)	12 (6.2, 3.6–10.6)	62 (8.9, 7.0–11.2)
It is/was a requirement or was recommended by government/Ministry of Health	10 (10.4, 5.8–18.1)	16 (7.0, 4.3–11.0)	23 (12.7, 8.6–18.3)	11 (5.7, 3.2–9.9)	60 (8.6, 6.7–10.9)
To prevent the possibility of complications of infection or severe disease	3 (3.1, 1.1–8.8)	39 (17.0, 12.5–22.0)	11 (6.1, 3.3–10.6)	2 (1.0, 0.3–3.7)	55 (7.9, 6.1–10.2)
The presence of chronic illness made me concerned about the effect of infection with the disease	1 (1.0, 0.2–5.5)	3 (1.3, 0.4–3.8)	1 (0.6, 0.1–3.1)	5 (2.6, 1.1–6.1)	10 (1.4, 0.8–2.6)
It is/was a requirement for traveling	0 (0.0, 0.0–3.9)	4 (1.7, 0.6–4.4)	2 (1.1, 0.3–4.0)	2 (1.0, 0.3–3.7)	8 (1.1, 0.5–2.3)
Others *	0 (0.0, 0.0–3.9)	1 (0.4, 0.1–2.4)	2 (1.1, 0.3–4.0)	1 (0.5, 0.1–2.9)	4 (0.6, 0.2–1.6)

* It was recommended by my parents/colleagues/friends, to protect colleagues, to be a role model for others. CI Confidence Interval.

**Table 3:** Reasons for incomplete or no uptake of hepatitis B vaccine among healthcare workers with partial or no hepatitis B vaccination, Kenya.

Why Haven’t You Received the Hepatitis B Vaccine/Why Didn’t You Complete Hepatitis B Vaccination Schedule?	Partial Uptake of Hepatitis B Vaccine N = 410 n (%, 95% CI)	Never Received Hepatitis B Vaccine N = 473 n (%, 95% CI)	Total N = 883 n (%, 95% CI)
Vaccine unavailability, i.e., not readily accessible	265 (64.6, 59.9–69.1)	243 (51.4, 46.9–55.8)	508 (57.5, 54.2–60.8)
Lack of knowledge about the vaccine	42 (10.2, 7.7–13.6)	212 (44.8, 40.4–49.3)	254 (28.8, 25.9–31.8)
Vaccine was not offered to me	121 (29.5, 25.3–34.1)	128 (27.1, 23.3–31.2)	249 (28.2, 25.3–31.3)
Cost of receiving the vaccine	81 (19.8, 16.2–23.9)	73 (15.4, 12.5–19.0)	154 (17.4, 15.1–20.1)
No follow-up for remaining doses	41 (10.0, 7.5–13.3)	0 (0.0, 0.0–0.8)	41 (4.6, 3.4–6.2)
The vaccine is not necessary	8 (2.0, 1.0–4.0)	21 (4.4, 2.9–6.8)	29 (3.3, 2.3–4.7)
Fear of the method of administration of the vaccine	4 (1.0, 0.4–2.5)	21 (4.4, 2.9–6.8)	25 (2.8, 1.9–4.2)
The vaccine is not mandatory	5 (1.2, 0.5–2.9)	17 (3.6, 2.3–5.8)	22 (2.5, 1.7–3.8)
Concerns about safety of the vaccine	5 (1.2, 0.5–2.9)	16 (3.4, 2.1–5.5)	21 (2.4, 1.6–3.7)
HCW availability constraints	12 (2.9, 1.7–5.1)	8 (1.7, 0.9–3.4)	20 (2.3, 1.5–3.6)
I do not consider myself at risk of contracting the disease	6 (1.5, 0.6–3.3)	12 (2.5, 1.4–4.4)	16 (1.8, 1.1–2.9)
Still within dosing schedule	9 (2.2, 1.1–4.1)	0 (0.0, 0.0–0.8)	9 (1.0, 0.5–1.9)
Concerns about receiving too many vaccines	0 (0.0, 0.0–0.8)	8 (1.7, 0.9–3.4)	8 (0.9, 0.5–1.8)
Lack of interest in the vaccine	0 (0.0, 0.0–0.8)	8 (1.7, 0.9–3.4)	8 (0.9, 0.5–1.8)
I have natural immunity against the disease	3 (0.7, 0.2–2.1)	4 (0.8, 0.3–2.2)	7 (0.8, 0.4–1.6)
Concerns about receiving the vaccine too many times	5 (1.2, 0.5–2.9)	2 (0.4, 0.1–1.7)	5 (0.6, 0.2–1.3)
I experienced an adverse event after receiving the vaccine	5 (1.2, 0.5–2.9)	0 (0.0, 0.0–0.8)	5 (0.6, 0.2–1.3)
Concerns about the effectiveness of the vaccine	0 (0.0, 0.0–0.8)	4 (0.8, 0.3–2.2)	4 (0.5, 0.2–1.2)
Fear of contracting the disease through vaccination	1 (0.2, 0.0–1.4)	3 (0.6, 0.2–1.8)	3 (0.3, 0.1–0.9)
Lack of knowledge on importance of the vaccine	3 (0.7, 0.2–2.1)	0 (0.0, 0.0–0.8)	3 (0.3, 0.1–0.9)
Lack of knowledge on vaccination schedule	3 (0.7, 0.2–2.1)	0 (0.0, 0.0–0.8)	3 (0.3, 0.1–0.9)
Other ^#^	0 (0.0, 0.0–0.8)	4 (0.8, 0.3–2.2)	4 (0.5, 0.2–1.2)

^#^ Religious reasons, no specific reason, fear of unknown. CI Confidence Interval.

## Data Availability

The datasets used and/or analyzed during the current study are available from the corresponding author on reasonable request.

## Supplementary Materials

The following supporting information can be downloaded at https://www.mdpi.com/article/10.3390/vaccines14100851/s1, Figure S1: Flow of healthcare workers included in the hepatitis B vaccination analysis, Kenya, 2023. Table S1: Distribution of enrolled healthcare workers by county, health facility level, and ownership type; Table S2: Focus group discussion guide; Table S3: Perceived ease of access to hepatitis B vaccine among participants enrolled in the study; Table S4: Multivariable analysis of factors associated with completion of the multidose hepatitis B vaccination schedule among healthcare workers in Kenya; Table S5: Multivariable mixed-effects logistic regression analysis of factors associated with adherence to multi-dose vaccination schedules for hepatitis B vaccine among HCWs enrolled in the study; Table S6: Multivariable mixed-effects logistic regression analysis of factors associated with adherence to multi-dose vaccination schedules for hepatitis B vaccine among healthcare workers enrolled in the study; Table S7: Multivariable mixed-effects logistic regression analysis of factors associated with adherence to multi-dose vaccination schedules for hepatitis B vaccine among healthcare workers enrolled in the study.
