# Supplementary material for: Completion of Hepatitis B Vaccination Schedule Among Healthcare Workers in Four Counties in Kenya: A Mixed-Methods Study
Source: Vaccines (Basel). 2026 Sep 27;14(10):851. doi: 10.3390/vaccines14100851 (PMC13709842; doi:10.3390/vaccines14100851)
Supplement: Supplementary file 1 [file vaccines-14-00851-s001.zip › vaccines-4545893-supplementary.pdf]

Supplementary Figure S1. Flow of healthcare workers included in the hepatitis B vaccination analysis, Kenya, 2023.

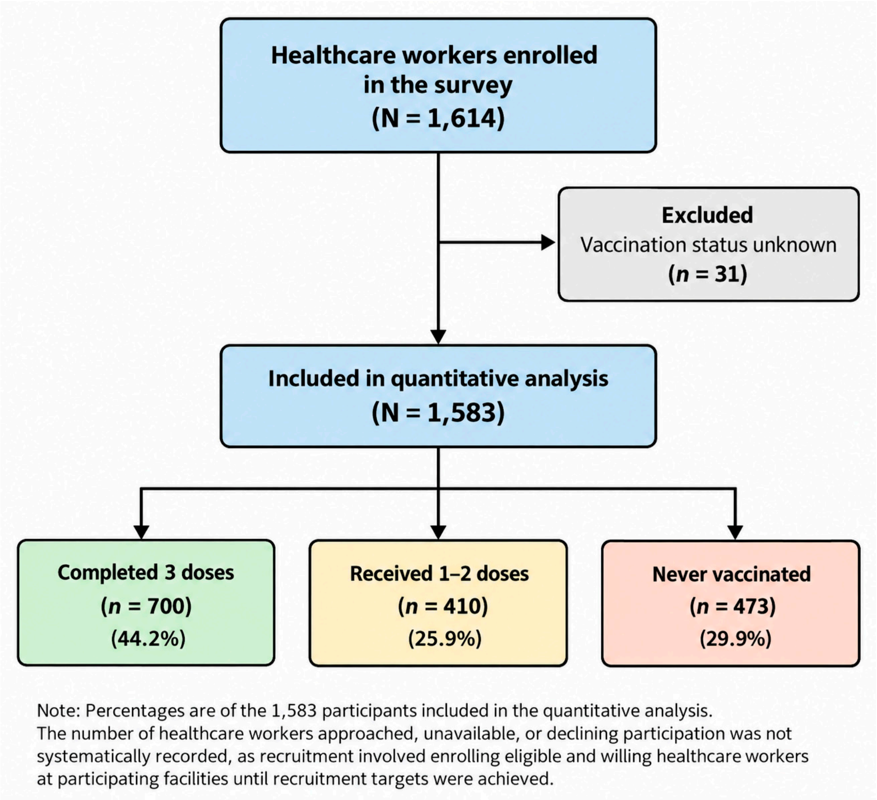

Supplementary Table S1: Distribution of enrolled HCWs by county, health facility level, and ownership type

Level	Kakamega			Nakuru			Mombasa			Siaya		
	Public	Private	Faith based	Public	Private	Faith based	Public	Private	Faith based	Public	Private	Faith based
2	54	13	1	45	21	2	38	27	2	85	15	1
3	40	11	6	46	7	5	10	7	0	102	21	16
4	80	0	0	37	50	23	10	15	0	158	147	0
5	85	0	0	175	0	0	103	156	0	0	0	0
**Total**		**290**			**411**			**368**			**545**	

Supplementary Table S2: Focus group discussion guide

Study site:
No. in a Group:
County:
FGD code No:

Themes	Main question	Probe if not captured
**General understanding**	1. What are some of the infectious risks that HCWs encounter at the workplace<br><br>2. What vaccines are recommended for HCWs?	What is the role of:<br>i. The employer in reducing these risks at the workplace?<br>ii. The HCW in reducing the risk of contracting diseases at the workplace?<br><br>iii. What are your general views on the vaccination of healthcare workers? Is it important/necessary?<br>iv. Probe more on influenza vaccine if it's mentioned
**Knowledge (access to information, gaps & modes of communication**	3. If you were looking for information related to vaccines for HCWs, where would you turn to or which resources would you turn to for the information?<br><br>4. What kind of information do you get from these sources<br><br>5. What would be the most appropriate mechanism for the MoH to inform HCWs about vaccines meant for them? Modes of communication	i. Where would you go to look it up?<br>ii. What sources of information related to vaccines do you think are most trustworthy, and why?<br><br>iii. Do you find the range of information useful?<br>iv. Is there certain information you find amiss?<br><br>v. What information platforms could be used to reach different cadres of HCWs?

**HCWs vaccination associated access and challenges**	6. Are there any challenges you face regarding vaccinations meant for healthcare workers at your workplace? [***Ask per vaccine i.e., Hepatitis B, COVID-19, typhoid, influenza***]<br>7. Do you feel there is proper documentation and follow up on vaccines recommended for HCWs	i. What are some of the cost-related challenges?<br>ii. What's your comment on [un]availability of vaccines?<br>iii. Adequacy (or lack of guidelines) on when to receive the vaccine?<br>iv. Why do you say so?
**Considerations before agreeing to take vaccines recommended for HCWs**	8. What factors would you consider before agreeing to take up vaccines that are recommended for HCWs by MoH?	i. Probe if not mentioned: The recommendation by MoH; the vaccine safety; perceived effectiveness; **frequency of administration,** perceived/known side effects, accessibility of vaccine
**Influenza characterization and references**	9. What is influenza?<br>10. How concerned are you about getting influenza?<br>11. What are some of the ways of preventing influenza?	i. What are some of the signs and symptoms of influenza virus infection?<br>ii. Why do you feel that way? How likely is it to happen? How severe do you think it would be?<br>iii. Probe more if they mention the influenza vaccine
**Attitudes**	12. What is your experience with influenza vaccination for HCWs?<br>13. What is your opinion about the recommendation by MoH for HCWs to receive the influenza vaccine?	i. What have you heard about other HCW's experiences with influenza vaccination?<br>ii. What do you think HCWs need to know in order to accept the influenza vaccine?<br>iii. Does it reduce the risk of transmitting the virus? How about the risk of being infected with the virus?<br>iv. How important is influenza vaccination?<br>v. Thoughts about safety of vaccine.

	14. In general, how do you feel about the influenza vaccine? [***Do you think there is a need to improve influenza vaccination trust and uptake among healthcare workers?]***	vi. Thoughts of whether it works or not.<br>iv. Have you heard about anything that worries you about the influenza vaccine? Who did you hear it from?
**Motivators to influenza vaccine uptake**	15. During the recent influenza vaccination exercise among HCWs, some HCWs received the vaccine. What factors made them take up the vaccine?	What were their reasons for receiving the vaccine? (**Probe;** self-protection, protection of patients, protection of family, work ethics, trust in the vaccine, recommendation by employer/ MoH, was offered for free, presence of chronic illness, the need to be a role model to other HCWs)

**Barriers to influenza vaccine uptake**	16. During the recent influenza vaccination exercise among HCWs, some HCWs did not receive the vaccine. Why do you think that happened?<br><br>17. How do you think this could be changed?<br><br>18. What are some of the missing information that still make HCWs hesitant to acquire the influenza vaccine?<br><br>19. Is the current training, sensitization and awareness on the influenza vaccine among HCWs sufficient to accelerate uptake?	i. What were their reasons? [**Probe;** availability of the vaccine & the timing of the influenza vaccination, belief that influenza is not a fatal disease and belief in host defense, doubts about vaccine efficacy and the fear of adverse reactions, dislike of injections and perceived too many vaccines for HCWs, religious beliefs, colleagues' experiences with influenza vaccine, fear of contracting disease through vaccination<br><br>ii. Probe for ways to overcoming barriers<br><br>iii. How do we overcome such awareness gap? How does this affect the uptake as per the government regulations among HCWs?<br><br>i. Is the awareness level sufficient enough to trigger influenza vaccination? Why do you say so?
**Recommendation for annual influenza vaccination among HCWs**	20. Before the influenza vaccine is introduced or HCWs throughout the country, what do you think HCWs would want to know about the vaccine?<br><br>21. Would you prefer if the vaccine is available throughout the year so that HCWs can take it at their own pace, or do you prefer if its available for a specific short period of time (campaign)?	i. What would be the best way to inform HCWs about influenza vaccine?<br><br>ii. Why do you say so?

	22. The MoH recommends annual vaccination of HCWs with the influenza vaccine. Would this be something you are willing to do?	iii. Probe for under what circumstances the HCWs are willing to receive the annual influenza vaccination.<br>iv. **If no**, probe for the reasons why they are not willing to receive the annual influenza vaccine

Supplementary Table S3: Perceived ease of access to hepatitis B vaccine among participants enrolled in the study (N=1583)

Characteristic	Complete uptake of hepatitis B vaccine N=700<br>n (%, 95% CI)	Partial uptake of hepatitis B vaccine N=410<br>n (%, 95% CI)	Never received hepatitis B vaccine N=473<br>n (%, 95% CI)	Overall N=1583<br>n (%, 95% CI)
**How easy is it to get/access to Hepatitis B vaccination services**				
Difficult/very difficult	332 (47.4, 43.7 - 51.2)	277 (67.6, 62.8 - 72.1)	353 (74.6, 70.4 - 78.5)	962 (60.8, 58.3 - 63.2)
Easy/Very easy	265 (37.9, 34.2 - 41.6)	82 (20.0, 16.2 - 24.2)	33 (6.9, 4.8 - 9.7)	380 (24.0, 21.9 - 26.2)
Neutral	103 (14.7, 12.2 - 17.6)	51 (12.4, 9.4 - 16.0)	87 (18.4, 15.0 - 22.2)	241 (15.2, 13.5 - 17.1)

χ^2 test p-value < 0.001

Supplementary Table S4: Univariable mixed effects logistic regression of factors associated with completion of the multi-dose hepatitis B vaccine schedule among HCWs enrolled in the study (N=1583) (Comparing completed vaccine schedule to partial uptake or no vaccine uptake combined)

Characteristic	Overall (N=1583)<br>n (%)	Completed hepatitis B vaccine schedule N=700<br>n (%)	Partial uptake or never received hep B vaccine N=883<br>n (%)	Odds ratio (95% CI)<br>n (%)	P-value
**Sex**					**<0.001***
Female	996 (62.9)	387 (55.3)	609 (69.0)	Ref	
Male	587 (37.1)	313 (44.7)	274 (31.0)	1.8 (1.4 - 2.3)	
**Age**					**0.007***
19-29	543 (34.3)	260(37.1)	283 (32.0)	1.1 (0.8 - 1.4)	0.505
30-39	614 (38.8)	278 (39.7)	336 (38.1)	Ref	
40-49	257 (16.2)	90(12.9)	167 (18.9)	0.6 (0.5-0.9)	0.004
>50	169 (10.7)	72 (10.3)	97 (11.0)	0.8 (0.6-1.2)	0.318
**Cadre**					**<0.001***
Nurse/midwife	517 (32.7)	281 (40.1)	236 (26.7)	Ref	
Allied health professionals	245 (15.5)	92 (13.1)	153 (17.3)	0.5 (0.4-0.8)	<0.001
Clinician	310 (19.6)	162 (23.1)	148 (16.8)	0.9 (0.7-1.2)	0.441
Support staff	278 (17.6)	63 (9.0)	215 (24.3)	0.2 (0.1-0.3)	<0.001

Laboratory staff	125 (7.9)	79 (11.3)	46 (5.2)	1.8 (1.2-2.8)	0.009
Administrative staff	108 (6.8)	23 (3.3)	85 (9.6)	0.3 (0.2-0.4)	<0.001
**Highest level of education**					**<0.001***
High school certificate or less	234 (14.8)	49 (7)	185 (21.0)	0.3 (0.2 - 0.4)	<0.001
Diploma	1,013 (64.0)	472 (67.4)	541 (61.3)	Ref	
Bachelor's degree	301 (19.0)	153 (21.9)	148 (16.8)	1.1 (0.4 - 1.4)	0.651
Post graduate degree	35 (2.2)	26 (3.7)	9 (1.0)	1.9 (0.8- 4.2)	0.138
**Number of years worked in healthcare**					**0.155**
0-5	774 (48.9)	323 (46.1)	451 (51.1)	Ref	
6-10	423 (26.7)	197 (28.1)	226 (25.6)	1.3 (0.9 - 1.7)	0.082
11-20	256 (16.2)	120 (17.1)	136 (15.4)	1.4 (0.9-1.9)	0.059
>20	130 (8.2)	60 (8.6)	70 (7.9)	1.0 (0.7 - 1.6)	0.892
**Level of physical contact with patients**					**<0.001***
Minimal contact	42 (2.7)	17 (2.4)	25 (2.8)	0.6(0.3-1.1)	0.093
Moderate contact	250 (15.8)	82 (11.7)	168 (19.0)	0.5 (0.3-0.6)	<0.001
Significant contact	1,291 (81.6)	601 (85.9)	690 (78.1)	Ref	
**Reported underlying chronic condition**					
Yes	199 (12.6)	83 (11.9)	116 (13.1)	0.9 (0.6 - 1.2)	0.447
No	1,384 (87.4)	617 (88.1)	767 (86.9)	Ref	
**Health facility ownership type**	N= 1581				**0.024***
Public	1,077(68.0)	440 (62.9)	637 (72.1)	Ref	
Private	435 (27.5)	233 (33.3)	202 (22.9)	1.8 (1.2 - 2.8)	0.007
FBO	69 (4.4)	26 (3.7)	43 (4.9)	1.5 (0.7 - 3.2)	0.298
**BMI**					0.621
Underweight (≤18.4)	28 (1.8)	12 (1.7)	16 (1.8)	1.1 (0.5 -2.5)	0.857
Normal (18.5 -24.9)	648 (40.9)	265 (37.9)	383 (43.4)	Ref	
Overweight (25.0 -29.9)	569 (35.9)	262 (37.4)	307 (34.8)	1.1 (0.9 -1.4)	0.501
Obese ≥ 30	338 (21.4)	161 (23.0)	177 (20.0)	1.2 (0.9 -1.6)	0.184
**Experienced adverse reactions following vaccination**					
Yes	248 (15.7)	117 (16.7)	131 (14.8)	1.1 (0.8 -1.5)	0.635
No	1,335 (84.3)	583 (83.3)	752 (85.2)	Ref	

**Rural-urban classification of sub counties where HCW works**					
Rural	790 (49.9)	261 (37.3)	529 (59.9)	Ref	
Urban	793 (50.1)	529 (59.9)	354 (40.1)	2.4 (1.7 -3.4)	**<0.001**[*]
**Health facility level**					
Primary health facilities	560 (35.4)	198 (28.3)	362 (41.0)	Ref	
Referral health facilities	1,023 (64.6)	502 (71.7)	521 (59.0)	1.4 (0.9 - 2.1)	**0.08**[*]
**County**[#]					**<0.001**[*]
Kakamega	281 (17.8)	96(13.7)	185 (21.0)	Ref	
Mombasa	362 (22.9)	230 (32.9)	132 (14.9)	3.4 (1.9 - 6.1)	<0.001
Nakuru	404 (25.5)	181 (25.9)	223 (25.3)	1.8 (1.1 - 3.1)	0.027
Siaya	536 (33.9)	193 (27.6)	343 (38.8)	1.2 (0.8 - 2.0)	0.429

[#]County was not included in multivariable model due to collinearity with rural-urban classification of sub counties

[*] Significant p values at p ≤ 0.25

Supplementary Table S5*: **<u>Multivariable mixed-effects logistic regression analysis of factors associated with adherence to multi-dose vaccination schedules for hepatitis B vaccine among</u> HCWs enrolled in the study (Comparing completed vaccine schedule to partial uptake or no vaccine uptake combined)***

Variable	aOR (95% CI)	P value
**Sex**		
Male	1.7 (1.4 - 2.2)	**<0.001**
Female	Ref	
**Age**		
19-29	1.3 (1.0-1.8)	0.086
30-39	Ref	
40-49	0.6 (0.4-0.9)	**0.022**
>50	1.0 (0.6-1.7)	0.978
**Cadre**		
Administrative staff	0.3 (0.2-0.4)	**<0.001**
Clinician	0.7 (0.5-0.9)	**0.037**
Nurses	Ref	
Laboratory staff	1.4 (0.9-2.2)	0.135
Allied health professionals	0.5 (0.3-0.7)	**<0.001**
Support staff	0.2 (0.2-0.4)	**<0.001**
**Years worked**		
0-5	Ref	
6-10	1.4 (1.0-2.0)	**0.034**
11-20	1.9 (1.2-3.0)	**0.004**
>20	0.9 (0.5-1.8)	0.937
**Rural-urban classification**		
Rural	Ref	
Urban	2.2 (1.5– 3.1)	**<0.001**

**Health facility level**		
Primary health facilities	Ref	
Referral health facilities	1.3 (0.9-1.9)	0.140
**Experienced adverse reactions following vaccination**		
Yes	1.1 (0.8-1.5)	0.508
No	Ref	

Supplementary Table S6: Multivariable mixed-effects logistic regression analysis of factors associated with adherence to multi-dose vaccination schedules for hepatitis B vaccine among healthcare workers enrolled in the study (Comparing completed vaccine schedule to partial uptake of hepatitis B vaccine dosing schedule)

**Variable**	**aOR (95% CI)**	**P value**
**Sex**		
Male	1.6 (1.2 - 2.1)	**0.003**
Female	Ref	
**Cadre**		
Administrative staff	0.7 (0.4 - 1.4)	0.292
Clinician	0.9 (0.7 - 1.4)	0.787
Nurses	Ref	
Laboratory staff	1.7 (1.0 – 2.9)	0.057
Allied health professionals	0.6 (0.4 – 1.0)	**0.028**
Support staff	0.6 (0.4 – 0.9)	**0.011**
**Rural-urban classification**		
Rural	Ref	
Urban	1.8 (1.2 - 2.7)	**0.002**
**Health facility level**		
Primary health facilities	Ref	
Referral health facilities	1.4 (1.0-2.1)	0.065
**Experienced adverse reactions following vaccination**		
Yes	1.1 (0.8-1.6)	0.604
No	Ref	

Supplementary Table S7: Multivariable mixed-effects logistic regression analysis of factors associated with adherence to multi-dose vaccination schedules for hepatitis B vaccine among healthcare workers enrolled in the study (Comparing completed vaccine schedule to never received hepatitis B vaccine)

**Variable**	**aOR (95% CI)**	**P value**
**Sex**		
Male	2.4 (1.8 - 3.3)	**<0.001**
Female	Ref	
**Cadre**		

Administrative staff	0.1 (0.06 - 0.2)	**<0.001**
Clinician	0.5 (0.3 - 0.8)	**0.002**
Nurses	Ref	
Laboratory staff	0.9 (0.5 - 1.7)	0.721
Allied health professionals	0.3 (0.2 - 0.5)	**<0.001**
Support staff	0.1 (0.06 - 0.14)	**<0.001**
**Number of years worked in healthcare**		
0-5	Ref	
6-10	1.6 (1.1-2.3)	**0.017**
11-20	1.2 (0.8-1.8)	0.444
>20	0.6 (0.4-1.1)	0.121
**BMI category**		
Underweight (≤18.4)	3.2 (0.9 - 11.4)	0.068
Normal (18.5 -24.9)	Ref	
Overweight (25.0 -29.9)	1.3 (0.9 - 1.8)	0.129
Obese ≥ 30	1.6 (1.1 - 2.5)	**0.020**
**Rural-urban classification**		
Rural	Ref	
Urban	2.8 (1.8-4.5)	**<0.001**
**Experienced adverse reactions following vaccination**		
Yes	1.1 (0.7-1.7)	0.647
No	Ref	
